# Tissue Regeneration in the Chronically Inflamed Tumor Environment: Implications for Cell Fusion Driven Tumor Progression and Therapy Resistant Tumor Hybrid Cells

**DOI:** 10.3390/ijms161226240

**Published:** 2015-12-19

**Authors:** Thomas Dittmar, Kurt S. Zänker

**Affiliations:** Institute of Immunology & Experimental Oncology, Center for Biomedical Education and Research (ZBAF), University of Witten/Herdecke, Witten 58453, Germany; kurt.zaenker@uni-wh.de

**Keywords:** cell fusion, tissue regeneration, cancer, mutator phenotype, mutations, aneuploidy, genomic instability, chromothripsis

## Abstract

The biological phenomenon of cell fusion in a cancer context is still a matter of controversial debates. Even though a plethora of *in vitro* and *in vivo* data have been published in the past decades the ultimate proof that tumor hybrid cells could originate in (human) cancers and could contribute to the progression of the disease is still missing, suggesting that the cell fusion hypothesis is rather fiction than fact. However, is the lack of this ultimate proof a valid argument against this hypothesis, particularly if one has to consider that appropriate markers do not (yet) exist, thus making it virtually impossible to identify a human tumor cell clearly as a tumor hybrid cell. In the present review, we will summarize the evidence supporting the cell fusion in cancer concept. Moreover, we will refine the cell fusion hypothesis by providing evidence that cell fusion is a potent inducer of aneuploidy, genomic instability and, most likely, even chromothripsis, suggesting that cell fusion, like mutations and aneuploidy, might be an inducer of a mutator phenotype. Finally, we will show that “accidental” tissue repair processes during cancer therapy could lead to the origin of therapy resistant cancer hybrid stem cells.

## 1. Introduction

Writing an article about the role of cell fusion in cancer usually starts with the following sentences: It is well recognized that the biological phenomenon of cell fusion plays a crucial role in several physiological events, like fertilization, placentation, skeletal muscle formation and wound healing/tissue regeneration, as well as pathophysiological processes including infection of host cells with enveloped viruses and cancer (an overview is given in [1,2]). Particularly, in cancer, cell fusion has been associated with disease progression due to numerous data providing evidence that hybrid cells derived from tumor cell × tumor cell or tumor cell × normal cell fusion events could exhibit novel properties, including an enhanced metastatogenic capacity, an increased proliferation rate, an enhanced drug resistance or a decreased apoptosis rate (an overview is given in [3,4,5]).

Even though there is a growing body of evidence demonstrating the impact of cell fusion in cancer progression this topic is still a matter of controversial debates. A plethora of animal studies have been done demonstrating that cell fusion is one mechanism how bone marrow-derived stem cells or cells of the myelomonocytic lineage could functionally adopt the phenotype of a foreign tissue [6,7,8,9,10,11,12,13,14]. Of course, the use of defined (transgenic) mice strains combined with sex-mismatch is advantageous in cell fusion studies and for the search of true hybrid cells, e.g., being identified as fully functional differentiated male cells within a female organ tissue environment, due to Cre-Lox mediated recombination resulting in target protein expression or due to an overlap of parental marker proteins. The latter strategy, for instance, was applied to demonstrate the fusion of macrophages and intestinal epithelial cells in a cancer context [15]. Thereby, a GFP mouse and an *APC^Min/+^*/ROSA26 mouse (also positive for β-Gal) were surgically joined (so-called parabiosis technique). After seven weeks, the mice were separated and the small intestine and the colon of the *APC^Min/+^*/ROSA26 mouse were analyzed revealing a significantly increased amount of cell fusion derived GFP and β-Gal double positive cells [15].

Unfortunately, the search for hybrid cells, particular in a cancer context, is much more difficult in humans, where cell fusion events could only be determined indirectly in tumor samples. Characteristics being associated with cell fusion, including aneuploidy or expression of, e.g., leukocyte markers on tumor cells, might be attributed to other “Non cell fusion” related mechanisms. Aneuploidy, a hallmark of most, if not all cancer cells [16,17], is potently induced by cell fusion [5,18,19], but could also be attributed to mutational alterations in proteins regulating the segregation of chromosomes during cell division [20,21,22]. Expression of, e.g., leukocyte markers on tumor cells might be related to fusion events with leukocytes, like macrophages [23,24], but might also be related to the aneuploidy induced genomic instability in tumor cells [25,26,27,28]. Likewise, hybrid cells may lose marker protein expression over time. This was reported by Powell and colleagues showing that murine macrophage × tumor cells lost the ability to express F4/80 protein, but retained mRNA expression [15].

Nonetheless, in the past years several papers about cell fusion in human cancer have been published, whereby different strategies have been applied to identify putative tumor cell × normal cell hybrids (Table 1).

**Table 1:** Identification of tumor hybrid cells in human cancers.

Authors	Tumor Cells	Normal Cells	Marker	Reference
Chakraborty *et al*., 2004	Renal cell carcinoma	BMDCs	PCR analysis of blood group alleles *	[23,29]
Yilmaz *et al*., 2004	Renal cell carcinoma	BMDCs	FISH analysis of Y chromosome *	[24,30]
Andersen *et al*., 2007	Multiple myeloma	Osteoclasts	FISH analysis	[31,32]
Shabo *et al*., 2008, 2009, 2011, 2013	Breast cancer, colorectal cancer	Macrophages	CD163, MAC387, DAP12	[33,34,35,36]
Clawson *et al*., 2012	Melanoma, colorectal cancer	Macrophages	CD45, CD14, Cytokeratin (KRT)	[24,29]
Lazova *et al*., 2013	Melanoma cells	BMDCs	STR analysis *	[37]
Ramakrishnan *et al*., 2013	Epithelial ovarian carcinoma	BMDCs	CD45, CXCR4	[23]
Clawson *et al*., 2015	Melanoma	Macrophages	CD14, CD68, CD163, CD204, CD206, CXCR4, CD44, ALCAM, MLANA	[32]

* Cancer patients that once received a BMT—Analysis of donor DNA in recipient cancer samples.

Expression of leukocyte antigens, like CD45 [23,24], CD163 [32,34,35] and DAP12 [33], on tumor cells has been considered as a putative proof for, e.g., macrophage × cancer cell hybrids. In fact, expression of CD163 on breast cancer cells as well as rectal cancer cells was associated with early recurrences and a reduced survival time [34,35], which is generally in view with the concept that tumor cell × normal cell hybrids may exhibit a more malignant phenotype [3,4,5].

More convincing data concerning hybrid cells found in human cancers were obtained from tumor samples of cancer patients that once had received a bone marrow transplant (BMT) [29,30,37]. For instance, analysis of tumor DNA by PCR from a metastatic renal cell carcinoma patient that once had received a BMT from his cancer-free brother revealed the presence of donor DNA in a lymph node metastasis suggesting hybridization between BMT cells and tumor cells [29]. Likewise, BMT donor Y chromosomes were identified by FISH analysis in a renal cell carcinoma of a female patient [30]. To date, the most persuasive data supporting that cell fusion events have occurred in a human cancer were published by Lazova and colleagues [37]. In this work, tumor cells free of leukocytes were isolated by laser microdissection from a brain metastasis of a male melanoma patient that had once received a BMT from his tumor-free brother. Short-tandem-repeat (STR) analysis of 14 different autosomal STR loci revealed an overlap of both donor and recipient alleles in all analyzed tumor cells suggesting that the brain metastasis was initiated by a clonal fusion event [37].

However, despite the increasing number of convincing “cell fusion in human cancer” articles there is still skepticism against this hypothesis since the ultimate argument that cancer cells could fuse with other cells and that the emerging tumor hybrid cells could foster tumor progression is still lacking. However, is the lack of this ultimate proof a valid argument against this hypothesis, particularly if one has to consider that appropriate markers do not (yet) exist thus making it virtually impossible to identify a human tumor cell clearly as a tumor hybrid cell?

In the present review, we will not only provide evidence that cell fusion events should frequently occur in human cancers, but we will also extend the current cell fusion hypothesis by demonstrating that cell fusion is a potent inducer of a mutator phenotype. Furthermore, we will discuss the relation between cell fusion and cancer therapy and the origin of drug resistant cancer hybrid cells.

### 1.1. Cell Fusion (in Human Cancer): Fact or Fiction?

The concept of cell fusion in cancer was postulated by the German physician Otto Aichel more than one century ago [38]. Aichel hypothesized that the fusion of leukocytes and tumor cells could be a mechanism how tumor cells could become aneuploid and could gain leukocyte properties like motility and to survive within the circulation. Within the past decades, a plethora of *in vitro* and *in vivo* studies verified Aichel’s visionary concept demonstrating that tumor cells could spontaneously fuse with tumor cells or other cells, thereby giving rise to hybrid cells exhibiting properties of both parental cells as well as novel properties (for an overview please refer to: [1,2,4,5,18,19,39,40]).

To understand why cell fusion events should commonly occur in cancer at all, one has to keep in mind that cell fusion plays a crucial role in wound healing and tissue regeneration (for review, see [1]). In fact, cell fusion has been demonstrated as one mechanism of how bone marrow-derived stem cells (BMDCs) and cells of the myelomonocytic lineage could restore organ tissue function and integrity [6,9,13,41,42,43,44]. However, the factors and conditions that will facilitate the fusion of two cells still remains to be elucidated, but inflammation has been identified as one positive trigger for cell fusion [45,46]. This is in view with recent published data that the pro-inflammatory cytokine TNF-α together with hypoxia, which is another common phenomenon of the tumor microenvironment, potently mediate the fusion of human breast epithelial cells and human breast cancer cells [47]. Similar findings were reported for the fusion of oral squamous carcinoma cells and endothelial cells, which was also positively triggered by TNF-α [48]. The causal link between inflammation and cell fusion is reasonable since inflammatory conditions are mandatory for the induction of the wound healing/tissue regeneration process [49,50].

It is well recognized that tumor tissue resembles chronically inflamed tissue and tumors are thus often referred to as “wounds that do not heal” [51,52,53]. Of particular importance in this context are tumor-associated macrophages (TAMs) and cancer-associated fibroblasts (CAFs), which are the key mediators of the chronically inflamed tumor microenvironment [54,55,56]. Secretion of cytokines, chemokines, growth factors, proteases and hormones by TAMs and CAFs will promote tumor progression due induction of neoangiogenesis, epithelial-to-mesenchymal transition (EMT), immune suppression and tumor cell proliferation [54,55,56]. Because of the sustained wound healing response in the chronically inflamed tumor microenvironment mediated by TAMs and CAFs it can be concluded that also cell fusion events will frequently occur. As mentioned above, cell fusion plays a crucial role in wound healing and tissue regeneration since this biological phenomenon represents one mechanism how, e.g., BMDCs could adopt tissue function of a foreign organ [6,9,13,41,42,43,44]. Likewise, inflammatory conditions *per se* or at least pro-inflammatory cytokines do foster cell fusion [45,46,47,48].

It thus remains ambiguous why the fact that cell fusion is involved in tissue regeneration is generally accepted, whereas cell fusion in cancer is not. All those cell types that have been demonstrated to regenerate normal organ tissue functionally by cell fusion will do the same with tumor cells since they do not discriminate between “good” tissue cells and “bad” tumor cells. Once the particular cell type received (a) defined signal(s), most likely initiated by inflammation, apoptosis and hypoxia [45,46,47,57], they will fuse with (a) damaged cell(s)—irrespective of whether the fusion partner will be a normal tissue cell or a tumor cell.

Because of that, it can be concluded that cell fusion events in human cancer are definitely real.

### 1.2. The Unpredictable and Random Nature of Cell Fusion; or: Is Cell Fusion an Inducer of the Mutator Phenotype?

A plethora of *in vitro* and *in vivo* data provided evidence that tumor cell × normal cell hybrids could exhibit novel properties including an increased metastatic capacity, an increased drug resistance or a decreased rate of apoptosis indicating the potency of such tumor hybrid cells in fostering tumor progression (for review, see [4,5,18,58]). Although these data are pretty convincing and, to us, definitely support the cell fusion in cancer hypothesis there is still some skepticism against the cell fusion in cancer hypothesis.

However, what is the ultimate argument that metastasizing or drug resistant cancer cells may not have originated once by cell fusion?

Why are the “mutator phenotype hypothesis” [59] or the “aneuploidy hypothesis” [17,60,61,62] more powerful assumptions to explain genomic instability and heterogeneity among tumor cells, which in turn has been suggested to be the main cause for the origin of metastatic or drug resistant tumor cells [60,63,64,65]. The “mutator phenotype” hypothesis [59,66] postulates that “mutator mutations” will result in an elevated mutation rate in tumor cells, which will lead to substantial genomic heterogeneity between the cell populations that will ultimately comprise a cancer [64]. Further mutations in driver genes will then give rise to genetically distinct lineages with similar phenotypes, but which constitute geographically distinct regions within the resulting cancer [64]. It was demonstrated that mutator mutations will have a greater effect if expressed early in carcinogenesis. The more mutations in a tumor, the more likely the tumor is associated with the expression of a mutator phenotype and that a mutator phenotype markedly increased the efficacy by which the tumor will acquire the multiple genetic changes required during tumorigenesis [64].

Li and Duesberg *et al*. postulated the “autocatalytic karyotype evolution” concept representing a dynamic model how aneuploidy will drive carcinogen-independent transformation of an “initiated” preneoplastic into a neoplastic cell [67]. Thereby, the degree of aneuploidy correlates with the malignancy of the disease (low degree of aneuploidy = low malignancy/primary tumor *vs*. high degree of aneuploidy = highly malignant/invasive and metastatic cancer) [67,68].

**Figure 1:**
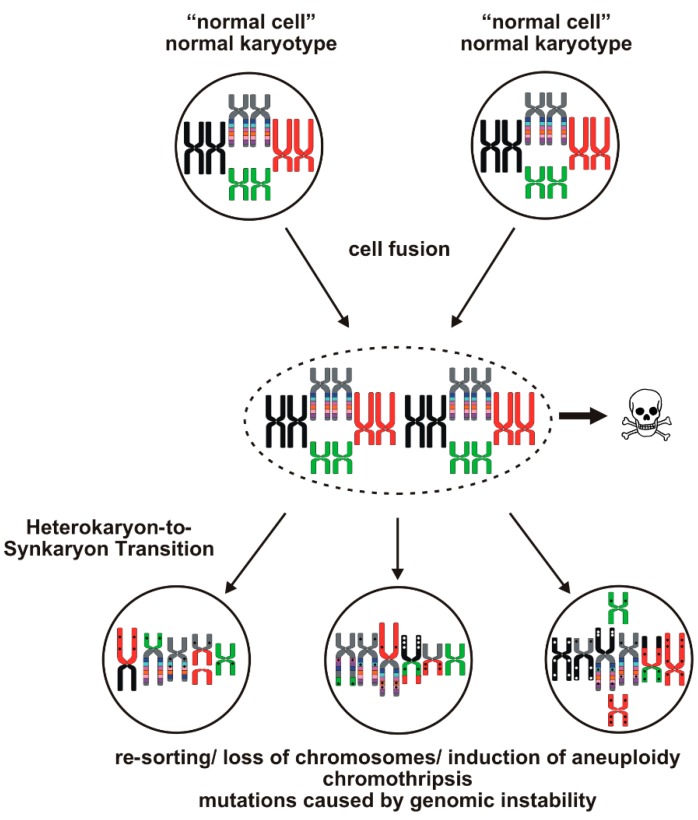
Induction of genomic instability by cell fusion. Fusion of two cells first result in heterokaryon being a hybrid cell with at least two distinct nuclei. The heterokaryon-to-synkaryon transition, representing the fusion of two parental nuclei to one hybrid nucleus, is a potent inducer of genomic instability in cells and is associated with a re-sorting, loss of chromosomes and putatively chromothripsis resulting in unique hybrid cells exhibiting an aneuploid karyotype and multiple mutations (indicated by small dots). However, most of the heterokaryons will die due to activation of tumor suppressor genes.

Both hypotheses (mutator *vs*. aneuploidy) coincide with being inducers of genetic variation and sustained genomic instability in tumor cells, thereby causing tumor tissue heterogeneity. However, various studies provided evidence that cell fusion is a potent inducer of genomic instability and aneuploidy in hybrid cells as well, which is chiefly attributed to the so-called heterokaryon-to-synkaryon transition [4,5,18,19,58,69,70]. The heterokaryon-to-synkaryon transition represents the fusion of the parental nuclei and is associated with a re-sorting (translocations, duplications, deletions), induction of aneuploidy and loss of whole chromosomes [70,71] (Figure 1).

In a recently published work, Zhou and colleagues demonstrated that cell fusion does connect oncogenesis with tumor evolution [71]. Here, non-transformed, cytogenetically stable rat intestinal epithelial cells were fused with each other resulting in hybrid cells possessing an unstable aneuploid karyotype concomitant with elevated rates of DNA damage including double-strand breaks, DNA fragmentation, and Robertsonian translocations [71]. Some of the evolved hybrid cell clones were highly tumorigenic in mice (tumor arose from 200 injected hybrid cells) and may thus further exhibit cancer stem/initiating cell capacities [71], which in turn would prove the assumption of Bjerkvig and colleagues that cancer stem cells could originate from cell fusion events [70].

The finding of elevated DNA fragmentation in hybrid cells [71] may point to chromothripsis [72] describing the phenomenon of multiple fragmentation and random re-arrangement of single and multiple chromosomes [72] (Figure 1). In tumor cells, chromothripsis has been shown to result in a loss of tumor suppressors, dysregulation of genes with known cancer links and amplification of oncogenes [72]. Interestingly, chromosome segments that fail to get reincorporated into the main chromosome(s) can circularize to become double minutes, which are frequently amplified [72]. In this regard, a study of Goldenberg et al. might be of interest demonstrating that the karyotype of hybrid cells, derived from human glioblastoma cells and hamster cells, showed the simultaneous presence of hamster-like, human-like, and a series of unidentifiable chromosomes [73] suggesting that these unidentifiable chromosomes may have originated from chromothripsis. Data of Jacobsen and colleagues revealed that about 30% of synkaryons, which derived from spontaneous fusion events between human breast adenocarcinoma cells and mouse stromal cells, had mixed mouse and human chromosome, among which 8% carried mouse/human translocations [74]. An increased number of unidentifiable chromosomes, which may have originated from chromothripsis, were also detected in radiation resistant and surviving multinucleated and giant cell phenotype (MNGC) [75]. Interestingly, cell fusion events induced senescence, high expression of senescence-associated secretory proteins (SASPs) and activation of pro-survival signals, like pAKT, BIRC3 and Bcl-xL, in MNGCs [75]. More importantly, MNGCs escaped senescence and despite having multiple spindle poles during mitosis, they overcame mitotic catastrophe to undergo normal cytokinesis forming mononucleated relapse population [75].

Another mechanism that has been associated with genomic instability and aneuploidy in cell fusion is the so-called phenomenon of “ploidy reduction” [76,77]. Ploidy reductions were first observed in fusion-derived polyploidy hepatocytes and represent one mechanism of how daughter cells with one-half chromosomal content could be generated [76,77]. Interestingly, ploidy reductions were associated with a loss of chromosomes in a non-random fashion suggesting that this mechanism could cause genomic instability and aneuploidy in daughter cells [76,77].

**Figure 2:**
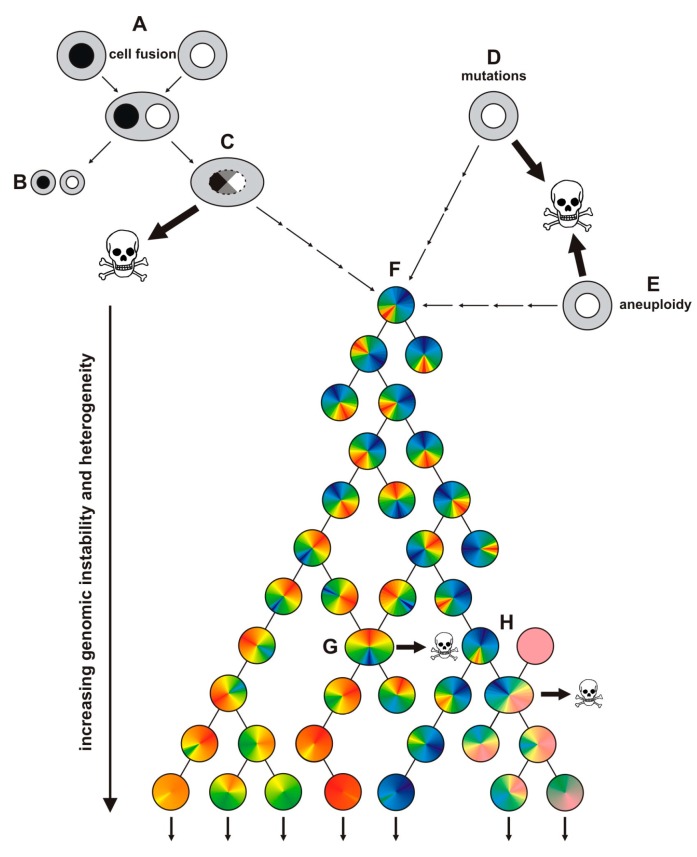
Induction of a mutator phenotype in cancer cells by cell fusion, mutation or aneuploidy. Cell fusion first result in heterokaryon formation (**A**); Such cells either remain in the heterokaryon state or could undergo ploidy reduction resulting in two daughter cells (**B**); However, in some cells cytogenesis might be defective causing the merging of the parental chromosomes (so-called heterokaryon-to-synkaryon transition) (**C**); Most of these hybrids will die because of activation of tumor suppressor genes, but a few cells will be able to survive and proliferate. Cell fusion, like mutations (**D**) and aneuploidy (**E**); is a potent inducer of genomic instability and could initiate a mutator phenotype in cancer cells (**F**) ultimately giving rise to phenotypically different genomic unstable cancer cells. Further cell fusion events could occur during tumor progression, whereby tumor cells and tumor cells (**G**) as well as tumor cells and other cells could fuse (**H**), thereby promoting the heterogeneity of the tumor tissue.

Thus, cell fusion could be a potent inducer of a sustained, self-autonomous genomic unstable phenotype in hybrid cells and which ultimately could lead to the malignant conversion of the cells. In that way, cell fusion resembles well to the “mutator phenotype hypothesis” and the “aneuploidy hypothesis”. Both hypotheses argue that due to “mutator mutations” or “aneuploidy” cancer cells with an elevated mutation rate or an increased susceptibility for aneuploidy events will originate, which will persist in the proliferating progenies, thereby giving rise to a huge population of genetically unstable cancer cells causing tumor tissue heterogeneity [60,64,67,68] (Figure 2).

**Figure 3:**
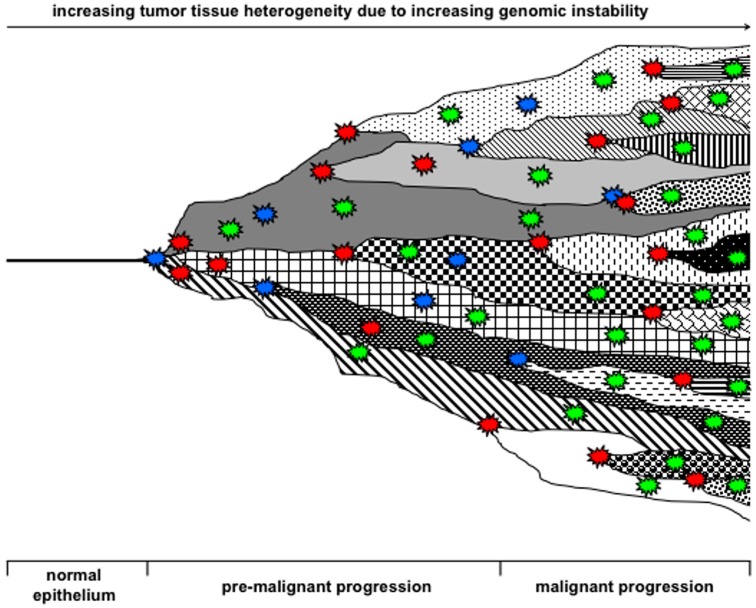
Model for tumor evolution and tumor tissue heterogeneity. In accordance with Figure 3, and to the interactive interplay of cell fusion, mutation and aneuploidy in causing a mutator phenotype the events were synonymously displayed as stars. Blue stars indicate mutator events, red stars indicate driver events and green stars indicate passenger events. The heterogeneity of the tumor tissue concomitant with the event increases in dependence of time and tumor progression, which also accounts for the number of mutator, driver and passenger events. Adapted from [64]. Cell fusion, mutation and aneuploidy are interacting with each other and are the real co-conspirators for induction of a mutator phenotype in cancer cells.

Thus, the provocative question will be: what is the difference between the “mutator phenotype hypothesis”, the “aneuploidy hypothesis” and the “cell fusion hypothesis” if one considers that all of them could lead to the malignant transformation of cells and all of them are potent inducers of a self-sustaining genomic instability. Because of these similarities we herewith postulate that all three processes rather work synergistically together and will influence each other both in the initiation and the progression of cancer; thus being the real co-conspirators and drivers of a mutator phenotype in cancer (Figure 3).

The relationship between mutations and aneuploidy has already been demonstrated in several studies, whereby mutations could either occur in genes coding for spindle or checkpoint proteins or in proteins regulating the expression of spindle or checkpoint proteins. For instance, overexpression of the mitotic arrest deficient 2 (Mad2) protein, being an essential component of the spindle checkpoint, is frequently found in human cancers and is associated with poor prognosis [78,79]. Overexpression of Mad2 in transgenic mice led to a variety of neoplasias, appearance of broken chromosomes, anaphase bridges and whole-chromosomes gains suggesting that elevated levels of Mad2 promotes aneuploidy and tumorigenesis in mice [21]. Since Mad2 mutations have not yet been identified it is assumed that altered Mad2 expression levels are rather attributed to p53 and pRB mutations, which both control Mad2 expression [80]. Transgenic mice expressing low levels of the spindle assembly checkpoint protein BubR1 developed progressive aneuploidy with a variety of progeroid features such as a short lifespan, cachectic dwarfism, cataracts or an impaired wound healing [81]. Interestingly, decreased expression levels of BubR1 and Bub1 were found to be epigenetically down-regulated in human carcinomas [82], indicating that, in accordance with Mad2, proteins regulating the expression of spindle checkpoint proteins might be altered. A similar mechanism may also account for the mitotic checkpoint protein Bub3 and its homologue Rae1. Cells from haplo-insufficient Rae1/Bub3 mice exhibited much greater rates of premature sister chromatid separation and chromosome missegregation than single haplo-insufficient cells [83]. Conjointly, haplo-insufficient Rae1/Bub3 mice were more susceptible to dimethylbenzanthrene-induced tumorigenesis than wild-type mice [83] demonstrating the relationship between aneuploidy and tumorigenesis. Mosaic variegated aneuploidy, being a rare recessive condition characterized by growth retardation, microcephaly, childhood cancer and a constitutional mosaicism for chromosomal gains and losses is attributed to truncating and missense mutations in the *BUB1B* gene, which encodes for the mitotic spindle checkpoint protein *BUBR1* [84]. This is in view with other data indicating that cancers displaying chromosomal instability were associated with a loss of function of the mitotic checkpoint protein BUB1 due to mutational inactivation [85]. In addition to checkpoint or spindle proteins, aneuploidy has also been associated with mutation inactivation of STAG2, a gene encoding a subunit of the cohesion complex, which regulates the separation of sister chromatids during cell division [20].

Aneuploidy itself has been demonstrated to be a potent driver of genomic instability and DNA damage in cells [25,27,60,86,87]. Studies in aneuploid yeast strains carrying extra copies of single chromosomes exhibited one or more forms of genomic instability including an increased chromosome loss and mitotic recombination as well as defective DNA damage repair [27]. Similar data were reported for non-transformed human retinal pigment epithelial (RPE-1) cells treated with Monastrol to induce aneuploidy by unequal chromosome segregation [88]. Albeit a DNA double-strand break response was initiated due to frequently damaged missegregated chromosomes during cytokinesis unbalanced translocations were identified in daughter cells [88], revealing the correlation of aneuploidy and genomic instability. Moreover, errors in mitotic chromosome segregation could generate DNA breaks and DNA damage via the formation of structures called micronuclei [87]. Tracking of such micronuclei revealed that they could undergo defective and asynchronous DNA replication resulting in DNA damage, like mutations, and often fragmentation of the chromosome in the micronucleus [87]. Interestingly, such micronuclei persisted in cells over several generations and chromosomes in the micronucleus were distributed to the daughter cells, which resembles well to the above mentioned phenomenon of chromothripsis [72] and the identification of unidentifiable chromosomes in hybrid cells [73,75].

As summarized above, cell fusion is a well-known inducer of genomic instability, aneuploidy [5,18,19,58,70], and thus mutations too. However, could both aneuploidy and mutations cause an increased frequency of cell fusion events? Unfortunately, to date considerably less is still known about the molecules and conditions that will promote the fusion of two cells [4,89]. Moreover, in dependence of the particular tissues cell fusion mechanisms do vary (for review, see [1,2]). For instance, the endogenous retroviral envelope protein syncytin-1 commonly facilitates the fusion of human trophoblasts to syncytiotrophoblasts [90], whereas in Drosophila the merging of fusion competent myoblasts is mediated by Arp2/3-dependent pathways that are required for actin dynamics for the podosome-like structures and fusion itself (for review, see [91]). Thus, because of the lack of knowledge of how the fusion of two cells is facilitated it is difficult to answer the question whether both aneuploidy and mutations may cause an increased frequency of cell fusion events. Nonetheless, a few papers have been published demonstrating that mutations and aneuploidy could alter the expression of cell fusion mediating proteins and could have an impact on cell fusion itself.

In a recently published work, Yu and colleagues demonstrated that syncytin-1 3’-LTR mutations (142T>C and 277A>G) were significantly associated with syncytin-1 overexpression in urothelial cell carcinomas [92] suggesting that mutations could result in the up-regulation of cell fusion associated molecules concomitant with an increased cell fusion frequency. Because syncytin-1 has also been associated with breast cancer cell fusion with endothelial cells [93] it would be interesting to investigate whether syncytin-1 expression in breast cancer cells might be related to a similar mechanism. Investigation of the importance of osteoactivin (OA/*GpnmB*) in osteogenesis using a transgenic mouse model with a nonsense mutation in the *GpnmB* gene revealed an increased osteoclastogenesis, which was attributed to an enhanced osteoclast differentiation and up-regulation of osteoclastic factors, like RANK receptors and DC-STAMP as well as stimulation of RANKL production by osteoblasts [94]. RANK receptors, RANKL and DC-STAMP are all involved in macrophage fusion (for review, see [91]). Studies of Silvestris and colleagues revealed that DC-STAMP might be involved in the fusion of osteoclasts and multiple myeloma cells thereby giving rise to myeloma polykaryons with bone-resorbing capacity [95]. An abnormal fusion and differentiation has been observed in cultured cytotrophoblasts isolated from trisomy 21 placentas revealing the aneuploidy could affect cell fusion [96]. Thereby, the impaired fusion capacity was related to an overexpression of the copper/zinc superoxide dismutase (SOD-1), encoded by chromosome 21, as well as an abnormal hCG signaling [96].

**Figure 4:**
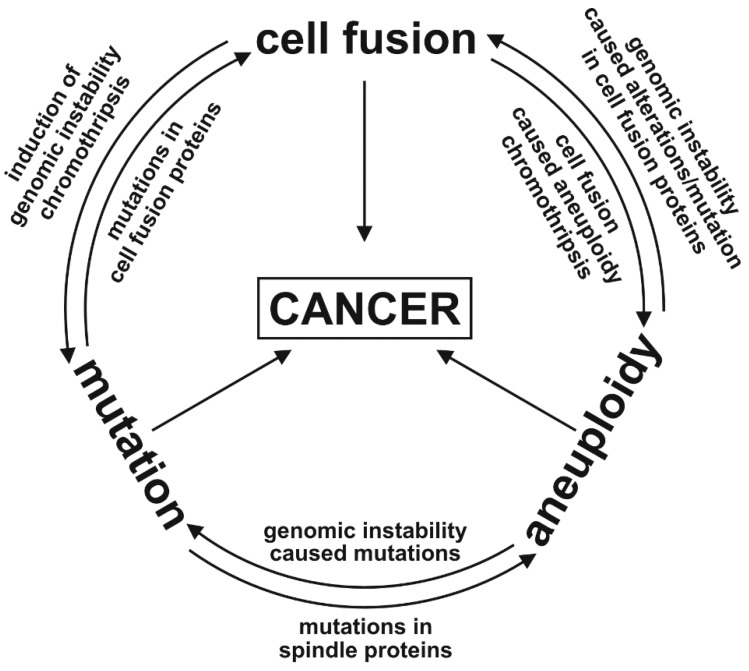
The interrelationship between mutations, aneuploidy and cell fusion as inducers of genomic instability and a mutator phenotype in cancer.

Even though only a few papers have been published so far demonstrating that fusion capacity of cells could be influenced in dependence of mutations and aneuploidy we are convinced that with the increasing knowledge about cell fusion mediating proteins and conditions more mutation and aneuploidy related effects modulating this biological phenomenon will be determined. The relationship between mutations, aneuploidy and cell fusion and how they could influence each other is schematically shown in Figure 4.

### 1.3. Evolution of Hybrid Cells Exhibiting Novel Properties by Cell Fusion?

The concept of “cell fusion in cancer” postulates that due to the fusion of tumor cells with other cells, like macrophages, fibroblasts, epithelial cells, etc. hybrid cells will evolve exhibiting novel properties including an enhanced metastatic capacity and/or an increased drug resistance (an overview is given in [3,4,5]). While this has been successfully validated in a plethora of studies (please refer to [15,23,32,57,74,95,97,98,99,100,101,102,103,104,105,106,107]) it remains questionable why cell fusion seems to be a more efficient mechanism to give rise to cells with novel properties than aneuploidy, particularly if it is considered that cell fusion itself is a potent inducer of aneuploidy. Moreover, cell fusion is an open, random process and because of that the ultimate phenotype of the evolving tumor hybrid cells cannot be predicted. Duelli and Lazebnik hypothesized that about 99% of the evolving tumor hybrid cells will remain in a quiescent state or will die, whereas the remaining 1% will survive, will proliferate and will exhibit novel properties [4]. However, due to the unpredictability of cell fusion, it cannot be ruled out that surviving and proliferating tumor hybrid cells will not exhibit novel properties or will be less malignant than the parental cancer cells. This, for instance, was demonstrated for hybrid cell clones derived from weakly malignant Cloudman S91 melanoma cells and murine macrophages [107]. Most of these hybrid clones showed an increased metastatic capacity, but a few hybrid clones not [107]. They exhibited a lower or even no metastatic capacity as compared to the parental melanoma cell line [107]. This is in view with recent findings of Zhou and colleagues studying the impact of cell fusion on oncogenesis [71]. Albeit most of the hybrid cell clones derived from non-transformed rat intestinal epithelial cells possessed a markedly increased tumor formation capacity some of the hybrid cell clones failed to initiate tumor growth in mice [71]. Likewise, we have demonstrated that hybrid clone cells, derived from spontaneous fusion events between human breast epithelial cells and human breast cancer cell lines, revealed a differential resistance to chemotherapeutic drugs [108]. While M13MDA435-4 hybrid clone cells showed an enhanced resistance towards doxorubicin and paclitaxel, M13MDA435-1 and M13MDA435-2 hybrid clone cells did not though all M13MDA435 hybrid clone cells evolved from the same cell fusion experiment [108].

However, it has to emphasized that the estimated amount of surviving hybrid cells of about 1% was determined in *in vitro* studies and thus the real number of surviving hybrid cells *in vivo* remains unknown—a matter that also accounts for the definite number of cell fusion events in cancer. Data obtained from animal studies and *in vitro* studies revealed that the fusion frequency in tumors was about 1% [4,105,109,110]. Thus considering a tumor of 1cm^3^ in size comprising of about 1 × 10^9^ cells and a fusion frequency and a survival rate of both 1% would result in 1 × 10^5^ proliferating tumor hybrid cells [4,105,109,110]. Interestingly, this estimated frequency of cell fusion events and surviving hybrid cells is very similar to the determined amount of aneuploidy-driven drug resistant (puromycin, colcemid, methotrexate) CHE cells, which was around 1–200 colonies per 10^6^ cells [111].

In any case, the finding that only 0.01% of the tumor cells will be tumor hybrid cells with new properties suggests that cell fusion events might be rather too rare to sufficiently explain the origin of, e.g., metastatic or drug resistance cancer cells. However, what is the argument that metastatic or drug resistant tumor cells do predominantly originate from non-cell fusion derived mutated and aneuploid cancer cells, particularly if it is considered that cell fusion is a potent inducer of genomic instability including aneuploidy and mutations? Are non-cell fusion derived aneuploid tumor cells harboring multiple mutations more potent to give rise to metastatic or drug resistant cancer cells than cell fusion derived aneuploid tumor hybrid cells harboring multiple mutations? Or is this argument simply of mathematical nature, namely that the probability that metastatic or drug resistant cancer cells will originate from non-fused cancer cells (representing 99.99% of the tumor mass, which is equal to 9.999 × 10^8^ of 1 × 10^9^ tumor cells) is higher in comparison to 0.01% tumor hybrid cells, which is equal to 1 × 10^5^ of 1 × 10^9^ tumor cells.

However, the exact number of cancer hybrid cells within a tumor still remains unknown. On the one hand this is attributed to the lack of appropriate markers clearly identifying a tumor cell as a tumor hybrid cell, which particularly accounts for tumor cell × tumor cell hybrids. Conjointly, cell fusion is a potent inducer of genomic instability and because of that tumor hybrid cells could lose marker expression by time. This, for instance, was demonstrated for macrophage × intestinal epithelial cell hybrids that lost F4/80 protein expression after a few weeks [15]. Zhou *et al*. revealed that cultivation of hybrid cells was associated with a markedly reduced mean chromosomal number indicating the dynamicity of cell fusion derived aneuploidy and genomic instability on a chromosomal level, which in turn does have a valid impact on the hybrid cells “omics” profile [71]. Furthermore, because of the chronically inflamed tumor microenvironment concomitant with a sustained healing/tissue regeneration process and the relationship between inflammation, wound healing and cell fusion, it can be concluded that the occurrence of cell fusion events in cancerous tissues is not a unique, but rather a persisting event suggesting that the real amount of tumor hybrid cells would be higher than 0.01%. This assumption is in view with recent data of Yan and colleagues demonstrating that about 6% of the tumor cells in a xenograft assay were identified as tumor hybrid cells [112]. Moreover, a higher amount of tumor hybrid cells of up to 12% were found in epirubicin treated mice [112] suggesting that certain conditions, like chemotherapy, could result in an increased fusion frequency.

However, irrespective of the real number of cancer hybrid cells in a tumor a general problem of the “cell fusion in cancer” concept might be a misinterpretation by concluding that, e.g., metastatic lesions or drug resistances will derive from just developed tumor hybrid cells, which definitely is not the case. It could be rather assumed that the origin of cell fusion-derived metastatic tumor hybrid cells or drug resistant is a long term process in accordance with the autocatalytic karyotype evolution model proposed by Li and Duesberg *et al*. [67]. Here Li and Duesberg and colleagues postulated that carcinogenesis is initiated by generating an aneuploid karyotype, which then destabilizes symmetric chromosome segregation due to unbalanced spindle and chromosomal proteins and centrosome numbers [67]. Aneuploidy then initiates autocatalytic variation and evolution that could give rise to new lethal preneoplastic and eventually neoplastic karyotypes [67], which, however, is in view with the above suggested concept that cell fusion could be a potent inducer of a mutator phenotype in cancer hybrid cells. Because of this relation it cannot be ruled out that the ancestors of metastatic or drug resistant cancer cells may have originated once by cell fusion.

### 1.4. Are Cancer Therapies Inducers of Cell Fusion and the Origin of Resistant Tumor (Hybrid) Cells?

A well-known phenomenon in cancer is the so-called oncogenic resistance describing the finding that recurrent cancers are frequently resistant to first-line therapy [113]. To us, and this has already been reviewed extensively [5,18,114], this phenomenon is contradictory to the cancer stem/initiating cell hypothesis postulating that cancer tissues are hierarchically organized like normal tissue comprising of a small number of (cancer) stem cell and a bulk of differentiated (tumor) cells [115,116]. Like cutting a dandelion off at ground level cancer stem/initiating cells will survive cancer therapies and will cause cancer recurrences months to years later [117,118]. The observation that recurrences commonly exhibit a more severe malignant phenotype and are resistant to first-line therapy indicate that the cancer stem/initiating cells causing a cancer relapse must be phenotypically different to those that originally induced the primary tumor. By using xenografting and DNA copy number alteration profiling of human BCR-ABL1 lymphoblastic leukemia cells Notta and colleagues demonstrated the existence of multiple genetically distinct leukemia-initiating cell subclones suggesting a branching multi-clonal model of leukemogenesis [119]. Interestingly, for some patient samples not the predominant, but rather minor subclones possessed repopulation capacities [119]. These data indicate that cancer stem/leukemia initiating cells are much more susceptible to chromosomal alterations resulting in the co-existence of genetically diverse subclones exhibiting unique repopulation capacities. Moreover, the findings of Notta and colleagues further indicate that cancer/leukemia therapy could result in environmental changes and growth conditions in the patient body enabling a minor subclone to become the repopulating clone.

It can be assumed that also solid cancers will harbor genetically diverse cancer stem/initiating cell clones, which originated via clonal evolution. Likewise, it cannot be ruled out that phenotypically diverse cancer stem/initiating cells may have originated independently from each other. This would be in view with findings indicating that Brac1 positive tumor cells in breast cancers contain distinct CD44^+^/CD24^−^ and CD133^+^ cells with cancer stem cell characteristics [120]. CD133 has also been used as a marker for colorectal carcinoma stem cells, whereas other studies demonstrated that such cells could be identified by expression of either EpCAM or CD44 or a combination of EpCAM, CD44 and CD166 or ALDH1 (for review, see [121]). The matter that cancer stem/initiating cells could be identified by differential markers does also belong to other solid tumors (for review, see [121]). However, since the different tumor stem/initiating cells were identified in various independent studies, it still remains unclear whether a solid tumor, like colorectal carcinoma, contains distinct populations of unique differential tumor stem/initiating cells or not.

Could cell fusion (in addition to mutations or aneuploidy) be a mechanism that could contribute to the origin of diverse cancer stem/initiating cell subclones and even leukemia initiating cells? Genomic analysis of the clonal origins of relapsed acute lymphoid leukemia was performed using a cohort of 47 B-progenitor ALL cells, of which six were classified as high hyperploid (>50 chromosomes) and 26 as low hyperploid (47–50 chromosomes) [122], suggesting that these leukemia initiating cells may have originated by cell fusion and subsequent aberrant ploidy reduction. This assumption would be in view with data of Skinner *et al*. providing evidence that cell fusion concomitant with subsequent ploidy reduction occurred between cells of the hematopoietic system under injury as well as non-injury conditions, whereby fusion events were identified in clonogenic progenitor as well as differentiated myeloid and lymphoid cells [123]. Moreover, Zhou and colleagues recently demonstrated that the number of chromosomes in fusion-derived clones near triploid or tetraploid at early passage, usually decreased with repeated passage resulting in hybrid cells having a near diploid karyotype of having >2N chromosomes [71]. These findings nicely illustrate the dynamicity of the loss of chromosomes, e.g., due to ploidy reductions, in tumor hybrid cells.

Rizvi and colleagues demonstrated that bone marrow-derived cells fused with normal and transformed intestinal stem cells [43]. It can be assumed that the fact that stem cells (either transformed or not) are targets of fusion events is of physiological relevance in order to ensure the status quo of the stem cell population and the overall homeostasis of a certain organ. It is well recognized that a lack of stem cells in a desired organ is associated with a loss of organ tissue integrity and homeostasis. Good examples of this relationship are bone marrow or hematopoietic stem cell transplantation studies. Without sufficient engraftment of transplanted hematopoietic stem cells the recipients will suffer from a non-reconstituted hematopoietic system [124].

As mentioned above macrophages (and other cells involved in tissue regeneration) do not discriminate between normal cells and tumor cells. Therefore, the same tissue regeneration program will run in cancer tissues [53] like in normal tissues. Because of this it would be worthwhile to speculate about the fate of cancer therapy treated primary tumor and metastatic tissues in the context of wound healing and tissue regeneration. Cancer therapies are generally targeting the rapidly proliferating tumor cells and the efficacy of the developed methods and therapeutic compounds is commonly measured by determining how fast and sufficient the tumors will shrink and what will be the benefit for the patients (disease free survival). However, elimination of cancer cells by anti-tumor therapy will cause a local inflammation, induced by tumor cell debris, concomitant with induction of a wound healing/tissue regeneration response including cell fusion events. This assumption is supported by recent data showing that radiation of glioblastoma cells resulted in the origin of a radiation resistant and surviving multinucleated and giant cell phenotype (MNGC). Interestingly, cell fusion events induced senescence, high expression of senescence-associated secretory proteins (SASPs) and activation of pro-survival signals, like pAKT, BIRC3 and Bcl-xL, in MNGCs [75]. More importantly, MNGCs escaped senescence and despite having multiple spindle poles during mitosis, they overcame mitotic catastrophe to undergo normal cytokinesis forming mononucleated relapse population [75]. However, even though parental cells and MNGCs showed similar modal chromosome numbers they not only markedly differed in the copy numbers of single chromosomes, but also in the presence of unidentifiable chromosomes/chromosomal structures which were found in relapse MNGCs [75] and which putatively originated by chromothripsis. As mentioned above, cell fusion could be a mechanism causing chromothripsis [71]. Moreover, in a recently published work Yan and colleagues demonstrated that chemotherapy could truly promote tumor cell hybridization *in vivo* [112]. Human SKBR3 breast cancer cells expressing two fluorescent marker proteins were used in xenograft studies and the tumors of those mice that were treated with epirubicin contained a higher amount of hybrid cells (12.2% ± 30%) as compared to controls (6.1% ± 1.6%) [112]. The finding that even in the control group hybrid cells were detected may point to an inherent fusion capacity of SKBR3 breast cancer cells, which has also been reported for melanoma cells [105] and which might be another proof that fusion events between tumor cells and tumor cells and normal cells do occur *in vivo*. Unfortunately, epirubicin-derived SKBR3 hybrids were not further analyzed and thus it remains to be elucidated whether hybrid cells do exhibit an altered karyotype and gene expression profile as compared to normal SKBR3 cells.

**Figure 5:**
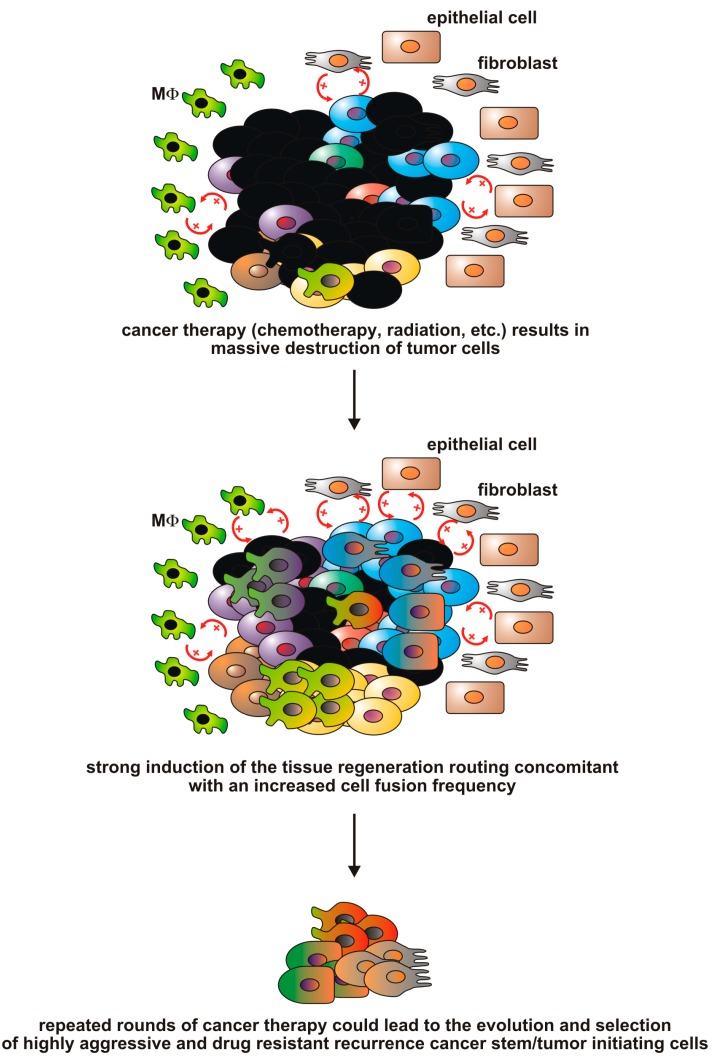
The causative correlation between cancer therapy and cell fusion events. Cancer therapy, e.g., chemotherapy and/or radiation therapy, will eliminate rapidly proliferating cancer cells. The accumulating tumor cell debris will initiate a local inflammation concomitant with induction of a macrophage driven wound healing/tissue regeneration response including cell fusion events. Repeating cycles and/or fractions of chemotherapy/radiation therapy could lead to the evolution and selection of cell fusion-derived recurrence cancer stem cells exhibiting a more malignant phenotype.

These data nicely illustrate that cancer therapy could result in the formation of cancer therapy resistant and relapse inducing multinucleated hybrid cells having a differential genomic background than their parental cells. Thus, cell fusion could be a mechanism that is associated with the origin of recurrence cancer stem cells (rCSCs) possessing an oncogenic resistance phenotype [114] (Figure 5). However, it should be emphasized that cell fusion is only one piece of this big puzzle since the event of cell fusion alone is not sufficient to explain the origin of rCSCs, but rather the mutational and aneuploidy events inside the hybrid cells that occur in consequence of cell fusion.

## 2. Conclusions

In the present work, we critically reviewed the role of cell fusion in cancer, which is still a matter of controversial debates. The general problems for this hypothesis in human cancers are the lack of appropriate markers to allow identifying real tumor hybrid cells (which would be the ultimate proof) and their amount in relation to normal non-fused derived tumor cells and whether such tumor hybrid cells would contribute to the progression of the disease at all. On the contrary, there is a growing body of evidence clearly indicating that cell fusion is a potent inducer of genomic instability [71,73,74,107,114,125], that hybrid cells could exhibit novel properties [15,23,32,57,74,95,97,98,99,100,101,102,103,104,105,106,107] and that cell fusion events in cancerous tissues do occur *in vivo* and could be induced by cancer therapy [15,24,32,33,34,35,43,112,125].

To us, all these data indicate that cell fusion in human cancer is a real phenomenon, which belong to both the initiation and the progression. As stated above, cell fusion is a potent inducer of genomic instability causing aneuploidy and multiple mutations in hybrid cells suggesting that cell fusion, like mutations and aneuploidy, is an inducer of a mutator phenotype in cancer cells. Because of that surviving cell fusion derived cancer hybrid cells harboring the mutator phenotype could give rise to phenotypically diverse and genomic unstable progenies of which some could ultimately initiate metastatic lesions or become drug resistant.

However, even though data of the recent years are pretty convincing and, to us, truly support the cell fusion in cancer concept, much more research has to be done in the upcoming years. This does not only include the search for appropriate cell fusion markers allowing for the detection and quantification of true cancer hybrid cells as well as the impact of such tumor hybrid cells in cancer, but also the search for factors and conditions that do trigger tumor cell hybridization with other cells. As mentioned above, considerably less is known about the molecules and conditions that will facilitate the fusion of two cells, including cancer cells. Inflammation, inflammatory cytokines, such as TNF-α, and apoptosis have already been identified as putative pro-fusogenic [45,46,47,48,57]. Likewise, the impact of cell fusion during cancer therapy has to be evaluated in future studies. Two recently published studies provided evidence that both radiation and chemotherapy could result in an increased fusion frequency and the origin of tumor hybrid cells that do have relapse capacity [75,112]. Even though it remains unclear whether these tumor hybrid cells will possess a putative rCSC phenotype [114], these studies indicate that cancer therapy could be associated with an increased fusion frequency of tumor cells, thereby giving rise to proliferating genomic unstable cancer hybrid cells.

In conclusion, cell fusion is becoming a reality in human cancers and could be a new target for novel anti-cancer therapies.
